# Correlation between serum lactate dehydrogenase, β_2_-microglobulin and free light chain levels and prognosis in patients with multiple myeloma

**DOI:** 10.12669/pjms.42.1.12581

**Published:** 2026-01

**Authors:** Yiyang Li, Sining Xing, Xiaoxue Huang, Yang Yu, Yankui Shi

**Affiliations:** 1 Yiyang Li, Department of Laboratory, Affiliated Hospital of Hebei University, Baoding 071000, Hebei, China; 2 Sining Xing, Department of Laboratory, Affiliated Hospital of Hebei University, Baoding 071000, Hebei, China; 3 Xiaoxue Huang, Department of Laboratory, Affiliated Hospital of Hebei University, Baoding 071000, Hebei, China; 4 Yang Yu, Department of Laboratory, Affiliated Hospital of Hebei University, Baoding 071000, Hebei, China; 5 Yankui Shi, Department of Laboratory, Affiliated Hospital of Hebei University, Baoding 071000, Hebei, China

**Keywords:** β_2_-microglobulin, Free light chain, Multiple myeloma, Lactate dehydrogenase, Prognosis

## Abstract

**Objective:**

To investigate the correlations between the serum levels of lactate dehydrogenase (LDH), β_2_-microglobulin(β_2_-MG) and free light chains (FLCs) and the prognosis of patients with multiple myeloma (MM).

**Methodology:**

This retrospective study included a total of 180 cases, among 80 patients diagnosed with MM(MM group) at Affiliated Hospital of Hebei University between January 2020 to January 2024 and 100 healthy individuals(control group) undergoing routine physical examinations during the same period. Prognosis was assessed using the Eastern Cooperative Oncology Group (ECOG) performance status scale. Serum levels of LDH, β_2_-MG and FLCs were compared among the three groups and the relationships between these biomarkers and the clinical characteristics of MM were analyzed.

**Results:**

Compared with the control group, the MM group exhibited significantly higher levels of LDH, β_2_-MG and FLCs(P< 0.05, respectively). In patients with MM, elevated serum levels of these biomarkers were significantly associated with advanced age(> 60 years), high ECOG scores(> 3) and advanced Durie-Salmon(DS) stage(stage III). Notably, significantly higher levels were observed in female patients, those with severe anemia and those with the immunoglobulin G IgG subtype of MM (P < 0.05, respectively). Patients in the PP group had significantly higher levels of LDH, β_2_-MG and FLCs compared with the FP group(P< 0.05, respectively).

**Conclusion:**

Elevated serum levels of LDH, β_2_-MG and FLC are risk factors for poor prognosis in patients with MM. Combined assessment of these three biomarkers may improve clinical evaluation and prognostic stratification.

## INTRODUCTION

Multiple myeloma (MM) is a hematologic malignancy characterized by the abnormal proliferation of plasma cells in the bone marrow, accounting for approximately 10% of all hematologic cancers. MM has diverse clinical manifestations, such as bone pain, anemia, renal impairment and hypercalcemia, all posing serious threats to patient health and survival.^1^ Despite significant advances in therapeutic strategies, such as proteasome inhibitors, immunomodulatory drugs and monoclonal antibodies, disease relapse and biological heterogeneity remain major challenges. A subset of patients continues to experience poor outcomes, highlighting the need for more accurate prognostic tools to support personalized treatment approaches.

Lactate dehydrogenase (LDH), β_2_-microglobulin (β_2_-MG) and serum free light chains (sFLC) are the most studied biomarkers in MM. With LDH being a key enzyme in glycolysis, elevated LDH levels indicate increased proliferative activity and metabolic load and have been shown in multiple studies to correlate with disease progression and poorer survival in patients with MM.^2^ β_2_-MG, a low-molecular-weight protein that forms part of the light chain of major histocompatibility complex class I molecules on the surface of most nucleated cells, is another important biomarker. Its serum concentration is associated with tumor burden and also serves as an indirect indicator of renal function, with renal impairment being a major risk factor for poor prognosis in MM.^3^ Free light chains (FLCs) are secreted by plasma cells and abnormal levels or ratios constitute one of the diagnostic criteria for MM. Compared with conventional electrophoresis, FLC assays offer improved sensitivity for detecting light-chain and oligosecretory MM, potentially identifying disease activity at earlier stages.^4^ While each of these markers has been individually validated in prognostic assessments, most prior studies have either focused on single biomarkers or integrated them with traditional staging systems.

Comprehensive analyses of their combined prognostic value remain limited. These biomarkers may reflect distinct yet complementary aspects of tumor biology: LDH indicates metabolic activity, β_2_-MG reflects tumor burden and renal function and sFLC captures early clonal plasma cell expansion. Therefore, a multi-marker approach could better capture disease heterogeneity. This study systematically evaluated the relationship between serum LDH, β_2_-MG and FLC levels and clinical prognosis in patients with MM, aiming to identify the potential utility of their combined detection in prognostic stratification and therapeutic decision-making.

## METHODOLOGY

This retrospective study included a total of 180 cases, among 80 patients diagnosed with MM who were treated at Affiliated Hospital of Hebei University between January 2020 to January 2024. Patients were rendered eligible for this study and assigned to the MM group. Additionally, data from 100 healthy individuals who underwent physical examinations during the same period were collected to serve as the control group. Clinical data were collected by members of the research team, including resident physicians and research assistants. The data included sex, age, presence of severe anemia, Eastern Cooperative Oncology Group (ECOG) performance status score, Durie-Salmon (DS) staging and immunoglobulin (Ig) subtype classification. Serum levels of LDH, β_2_-MG and FLCs at the time of admission were also recorded.

### Ethical approval:

The study was approved by the Institutional Ethics Committee of Affiliated Hospital of Hebei University(No.: HDFYLL-KY-2022-020; Date: December 30, 2022) and written informed consent was obtained from all participants.

### Inclusion criteria:

- Diagnosis consistent with the criteria outlined in the Specification of Diagnosis and Therapeutic Effect Evaluation of Blood Disease.^5^
- Pathological confirmation of MM.
- Complete clinical data.

### Exclusion criteria:

- Severe dysfunction or organic lesions of vital organs.
- Acute episodes of inflammatory diseases or adverse cardiovascular events upon admission.
- Concomitant malignancies, or other hematologic or hematopoietic malignancies.
- Anemia, immune dysfunction, or coagulopathy due to causes unrelated to MM.

### Prognostic Assessment:

Prognostic grouping of the 80 patients with MM was performed based on the ECOG score. Patients with an ECOG score > 3 (*n =* 22) were classified into the poor prognosis (PP) group and those with a score ≤ 3 (*n =* 58) were placed in the favorable prognosis (FP) group. In terms of the ECOG scale, performance status was rated on a 6-point scale: 0: Fully active, no performance restrictions;

- Restricted in physically strenuous activity but ambulatory;
- Ambulatory and capable of all self-care but unable to carry out work activities; up and about more than 50% of waking hours;
- Capable of only limited self-care; confined to bed or chair for more than 50% of waking hours;
- Completely disabled; cannot carry on any self-care; totally confined to bed or chair;
- Deceased.

### Statistical analysis:

All statistical analyses were performed using SPSS22.0. The confidence interval was 95%, measurement data were expressed as mean ± standard deviation (*χ̅*±*S*) and enumeration data as frequency and percentage (*n*[%]). Comparisons between groups were performed using either the *t*-test or the chi-square (χ²) test. A *P*-value< 0.05 was considered statistically significant. Logistic regression analysis was used to identify independent prognostic factors in patients with MM. Receiver operating characteristic (ROC) curves were plotted to evaluate the predictive performance of LDH, β_2_-MG and sFLC levels. A *P*-value < 0.05 was considered statistically significant.

## RESULTS

The levels of serum LDH, β_2_-MG and FLCs were significantly higher in the MM group than in the control group (*P <* 0.05, respectively) (Table-I). In patients with MM, serum levels of LDH, β_2_-MG and FLC were significantly associated with various clinical characteristics. All three markers increased with age (> 60 years), higher ECOG scores (> 3) and advanced DS stage (stage III). Additionally, their levels were significantly higher in female patients, those with severe anemia and those with the IgG subtype of MM (*P <* 0.05, respectively). These findings suggest that LDH, β_2_-MG and sFLC may play distinct roles in the pathogenesis, progression and subtype differentiation of MM. Combined detection of these markers may contribute to comprehensive disease and prognostic assessment (Table-II).

**Table-I:** Comparison of LDH, β₂-MG and sFLC levels between patients with MM and healthy controls (*χ̅*±*S*).

Group	Number of Cases (n)	LDH (U/L)	β₂-MG (μg/L)	sFLC (mg/L)
MM group	80	250.69±32.39	4.83±1.62	95.28±22.14
Control group	100	146.60±21.34	1.88±0.74	10.14±3.35
*t*		25.878	16.191	37.940
*P*		<0.001	<0.001	<0.001

**Table-II:** Association of serum LDH, β₂-MG and FLC levels with clinical characteristics in patients with MM (*χ̅*±*S*).

Biomarker	Category	LDH (U/L)	t/F	P	β₂-MG (μg/L)	t/F	P	sFLC (mg/L)	t/F	P
Sex	Male (n = 48)	229.09±13.88	12.743	<0.001	4.14±1.39	5.472	<0.001	86.55±14.38	4.912	<0.001
	Female (n = 32)	283.10±23.99			5.88±1.38			108.38±16.00		
Age	≤ 60 (n = 46)	228.06±13.24	12.542	<0.001	4.01±1.25	6.517	<0.001	85.80±13.83	5.118	<0.001
	> 60 (n = 34)	281.31±24.36			5.95±1.39			108.11±24.85		
Severe anemia	Present (n = 18)	300.41±17.60	13.249	<0.001	6.58±1.37	6.317	<0.001	118.58±23.67	6.137	<0.001
	Absent (n = 62)	236.26±18.21			4.42±1.32			88.51±16.49		
ECOG score	≤3 (n = 52)	231.21±15.25	12.878	<0.001	4.23±1.38	5.203	<0.001	86.55±13.99	5.674	<0.001
	>3 (n = 28)	286.88±23.29			5.95±1.46			111.49±25.40		
DS staging	Stage I–II (n = 48)	229.09±13.88	12.743	<0.001	4.14±1.39	5.472	<0.001	86.55±14.38	4.912	<0.001
	Stage III (n = 32)	283.10±23.99			5.88±1.39			108.38±25.31		
Ig subtype	IgA (n =25)	218.16±7.45	8.218	<0.001	3.99±1.15	3.320	0.001	82.59±11.55	3.727	<0.001
	IgG (n = 55)	265.48±28.25			5.22±1.67			101.05±23.44		

Compared with the FP group, the PP group showed significantly higher serum levels of LDH, β_2_-MG and FLC (*P <* 0.05, respectively) (Table-III). Logistic regression analysis was conducted using prognosis (poor vs. favorable) as the dependent variable and LDH, β_2_-MG and sFLC levels as independent variables. Results indicated that elevated serum LDH, β-MG and FLC levels were independent risk factors for poor prognosis in patients with MM (*P <* 0.05, respectively) (Table-IV and Table-V).

**Table-III:** Comparison of LDH, β₂-MG and sFLC levels between PP and FP groups (*χ̅*±*S*).

Group	Number of Cases (n)	LDH (U/L)	β₂-MG (μg/L)	sFLC (mg/L)
FP group	58	239.43±25.08	4.24±1.20	87.50±12.64
PP group	22	280.40±31.09	6.41±1.58	115.81±28.32
*t*	-	6.098	6.600	6.199
*P*	-	<0.001	<0.001	<0.001

**Table-IV:** Variable definitions for logistic regression analysis.

Definition	
Prognosis	1 = Poor, 0 = Favorable
LDH	Continuous variable (measured value)
β₂-MG	Continuous variable (measured value)
sFLC	Continuous variable (measured value)

**Table-V:** Logistic regression analysis of prognostic factors in patients with MM.

Variable	B	SE	Wald	P-value	OR	95% CI	
LDH	0.048	0.011	18.049	<0.001	1.049	1.026	1.072
β₂-MG	1.256	0.334	14.174	<0.001	3.512	1.826	6.753
sFLC	0.069	0.017	16.320	<0.001	1.072	1.036	1.108

***Note:*** SE - standard error; OR – odds ratio; 95% CI – 95% confidence interval.

**Fig.1:**
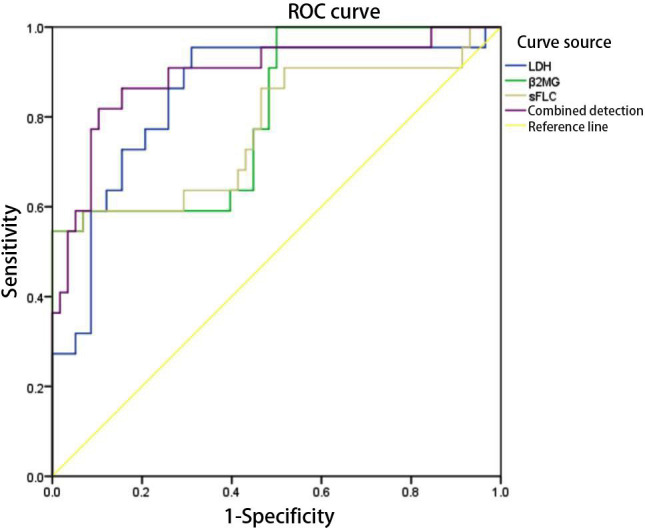
ROC curves of LDH, β_2_-MG and sFLC for predicting poor prognosis in patients with MM.

Receiver operating characteristic (ROC) curve analysis showed that LDH, β_2_-MG and sFLC individually predicted poor prognosis with areas under the curve (AUC) of 0.850, 0.806 and 0.775, respectively, while the combined model yielded an AUC of 0.893 (Table-VI and Fig.1).

**Table-VI:** ROC curve analysis of LDH, β₂-MG and sFLC for predicting poor prognosis in patients with MM.

Marker	AUC	SE	P-value	95% CI		Cut-off value	Youden index	Sensitivity (%)	Specificity (%)
				Min.	Max.				
LDH	0.850	0.051	<0.001	0.750	0.950	247.87	0.645	95.5	69.0
β₂-MG	0.806	0.056	<0.001	0.697	0.916	5.940	0.545	54.5	100.0
sFLC	0.775	0.067	<0.001	0.644	0.906	119.255	0.545	54.5	100.0
Combined detection	0.893	0.045	<0.001	0.805	0.982	-	0.715	81.8	89.7

***Note:*** SE – standard error; 95% CI – 95% confidence interval.

## DISCUSSION

The present study found significantly elevated serum levels of LDH, β_2_-MG and FLC in patients with MM compared with healthy controls. LDH is a key enzyme in cellular energy metabolism and its elevated levels are generally associated with active cell proliferation and tissue damage. This finding is consistent with the study by Buresova M et al.^6^, which demonstrated a correlation between circulating tumor DNA and LDH levels in patients with advanced non-small cell lung cancer. This suggests that the elevated LDH in patients with MM is likely attributed to the high proliferative activity of malignant plasma cells. β_2_-MG is the light chain component of the class I major histocompatibility complex on cell surfaces and its serum concentration is associated with tumor burden and disease activity.^7^,^8^ This observation aligns with the findings of Kim DK et al.^9^, whose use of a whole-body magnetic resonance imaging scoring system indirectly supported the association between β_2_-MG levels and tumor load. As the monomeric form of immunoglobulins, sFLC is another biomarker closely related to the diagnosis and disease monitoring of MM.^10^ The current findings reinforce the established role of sFLC in MM clinical assessment.

MM is a malignant tumor originating from plasma cells, characterized by the secretion of monoclonal immunoglobulins or their fragments by abnormally proliferating plasma cells in the bone marrow.^11^ Moreover, LDH, β_2_-MG and sFLC levels were strongly associated with key clinicopathological features among patients with MM. All three markers tended to be higher in patients over 60 years old, those with high ECOG scores (> 3) and those in stage III according to the DS staging system. Additionally, these markers were significantly elevated in female patients, those with severe anemia and those with the IgG subtype (*P <* 0.05, respectively). Jin X et al.^12^ found that high-risk cytogenetic abnormalities (*e.g*., 1q21 amplification) significantly affect the survival outcomes of patients with primary plasma cell leukemia, further demonstrating the critical role of genetic factors in MM progression.

These findings suggest that each of these biomarkers may play distinct roles in the pathogenesis, progression and subtype differentiation of MM. Combined detection of LDH, β_2_-MG and sFLC may facilitate a more comprehensive evaluation of disease status and prognosis. With increasing age, the decline in DNA repair capacity and accumulation of genetic mutations may promote the malignant proliferation of plasma cells. Chen H et al.^13^ reported that 1q21 amplification and elevated IL-21 levels were associated with poor prognosis in patients with MM, supporting the role of genomic instability in disease progression among elderly individuals. Additionally, increased secretion of cytokines such as IL-6 by bone marrow stromal cells in older patients may further stimulate tumor cell growth. T-cell dysfunction contributes to enhanced immune evasion and increased tumor burden, consistent with the findings of Zhai M et al.^14^, who reported a positive correlation between β_2_-MG levels and tumor cell count. Skeletal destruction (*e.g*., fractures of the lumbar spine and ribs) leads to restricted mobility, while renal impairment (induced by light chain deposition) causes fatigue and anemia (due to suppression of bone marrow hematopoiesis) exacerbates physical decline.

When myeloma cells constitute more than 30% of bone marrow cellularity and are accompanied by osteolytic lesions or hypercalcemia, LDH serves not only as a biomarker of renal injury but also as an indicator of the increased metabolic activity of tumor cells. This observation, as demonstrated by Reeves Sharma D et al.^15^, is closely linked to cell cycle dysregulation driven by genetic mutations. Zhang J et al.^16^ found that β_2_-MG levels were positively correlated with both the number of myeloma cells and the degree of renal impairment, further validating its role as a marker of tumor burden. When the synthesis of light chains exceeds that of heavy chains, the resulting unbound free light chains are released into circulation and tend to deposit in renal tubules.

Tao X et al.^17^ reported that combined detection of serum LDH, serum ferritin and sFLC significantly improves the diagnostic efficacy for MM. In cases of high clonal IgG production, patients are more susceptible to hyperviscosity syndrome (associated with elevated LDH) and renal impairment (elevated β_2_-MG), while sFLC levels may remain relatively low due to preserved heavy chain synthesis. A study by Zhu G et al.^18^ comparing different FLC detection systems indirectly supports the notion that the immunoglobulin subtype influences disease manifestation. These findings also suggest that the hormonal milieu may regulate the tumor microenvironment via IL-6 and other cytokines. As a key enzyme in glycolysis, LDH elevation reflects the heightened metabolism of tumor cells and is linked to uncontrolled cell cycle progression induced by oncogenic mutations. β_2_-MG, secreted by tumor cells, is directly associated with both tumor burden and renal impairment. Overproduction of light chains in the absence of adequate heavy chains leads to the release of unbound light chains into the bloodstream, which can deposit in the renal tubules and subsequent renal injury.

Furthermore, the logistic regression analysis in this study also identified high serum levels of LDH, β_2_-MG and FLC as independent risk factors for poor prognosis in MM. LDH, in particular, typically reflects active tumor metabolism and rapid proliferation. Gao D et al.^19^ confirmed that elevated LDH is closely associated with increased tumor burden, indicating a larger number of tumor cells in the body, consistent with the current study’s conclusion that LDH is a significant marker of poor prognosis. Increased LDH is associated with a greater tumor burden, indicating a larger number of tumor cells in the body.

Wang J et al.^20^ reported that elevated LDH levels were significantly associated with treatment response and prognosis in patients with newly diagnosed MM, providing further evidence for LDH’s clinical value as a marker of tumor burden. Since renal impairment is commonly found in patients with MM, an elevated β_2_-MG level may suggest renal involvement due to tumor progression. Wang SY et al.^21^ reported a positive correlation between β_2_-MG levels and DS staging as well as disease activity, with higher levels often indicating a poor prognosis in MM, which is consistent with this study’s conclusion that β_2_-MG is an independent prognostic risk factor. β_2_-MG not only reflects renal function but is also closely linked to tumor cell proliferation and disease activity, making its elevation a valuable indicator of a poor prognosis.

Cao D et al.^22^ showed that increased sFLC levels were significantly associated with disease progression and poor prognosis in patients with MM, supporting this study’s finding that sFLC is an independent risk factor for prognosis. In addition, sFLC levels also reflect the immune evasion capability of myeloma cells. Further, Leung N et al.^23^ confirmed that changes in sFLC levels could predict prognosis in patients with MM and related renal injury, suggesting a close link between sFLC levels, tumor-induced immunosuppression and disease progression. In patients with MM, elevated sFLC levels may be associated with tumor-induced immunosuppression, which impairs the body’s ability to produce an effective anti-tumor response, thereby accelerating disease progression and worsening prognosis. The combined detection of LDH, β_2_-MG and sFLC provides more comprehensive information. These biomarkers reflect the tumor’s metabolic activity, renal function and immune status from different perspectives, allowing clinicians to assess disease severity and progression more accurately. This, in turn, facilitates the development of personalized treatment strategies and more precise prognostic evaluations.

### Limitations:

This study is a retrospective single center study with a small sample size, and the results obtained are limited. It requires the participation of multiple centers and the expansion of the sample size to obtain more comprehensive results. More clinical trials are needed to verify its clinical efficacy and safety, and to analyze the factors that affect patient survival prognosis.

## CONCLUSIONS

LDH, β_2_-MG and sFLC are closely associated with the clinical and pathological characteristics of MM by reflecting different dimensions of the disease, including proliferative activity, tumor burden, plasma cell differentiation abnormalities and renal impairment. Their combined detection of LDH, β_2_-MG and sFLC enables a more thorough evaluation of disease severity and prognosis, thus providing a valuable reference for individualized treatment planning.

### Authors’ Contributions:

**YL, SX:** Carried out the studies, participated in collecting data and drafted the manuscript and are responsible and accountable for the accuracy or integrity of the work.

**XH, YY and YS:** Performed the statistical analysis and participated in its design. Critical Review.

All authors have read and approved the final manuscript.
